# Energy-Efficient Insulating Geopolymer Foams with the Addition of Phase Change Materials

**DOI:** 10.1021/acsomega.4c06227

**Published:** 2025-01-17

**Authors:** Joanna Marczyk, Agnieszka Przybek, Kinga Setlak, Patrycja Bazan, Michał Łach

**Affiliations:** Cracow University of Technology, Faculty of Materials Engineering and Physics, Warszawska 24, 31-155 Kraków, Poland

## Abstract

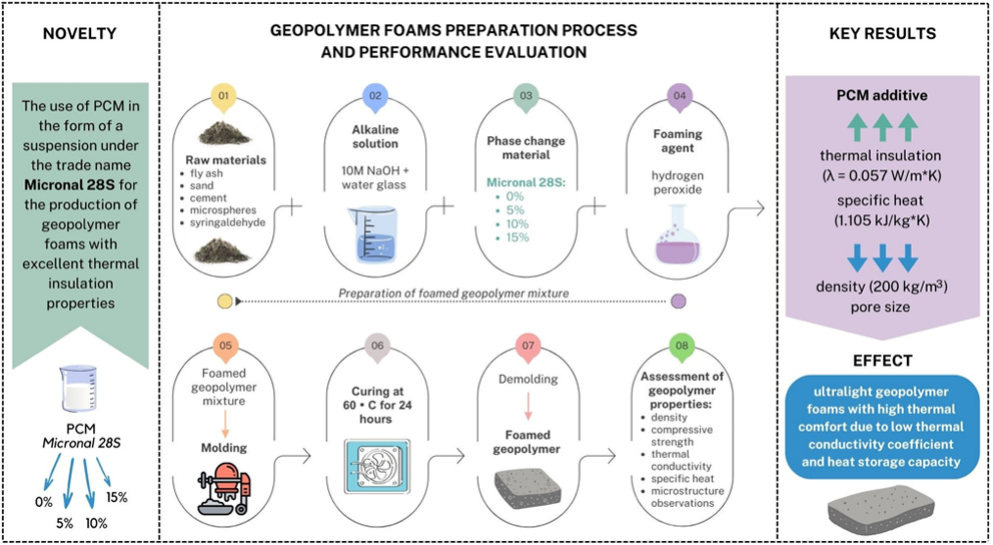

This study assessed the impact of the addition of phase change materials (PCMs) under the trade name Micronal 28S on the properties of the manufactured geopolymer foams. Micronal 28S is used as a functional component in foams (insulation materials), foamed building materials, and materials for temperature regulation to improve thermal comfort and indoor climate. The melting point of Micronal 28S is 28 ± 2 °C, and the heat of fusion is ∼140 J/g. As part of the research, geopolymer mixtures containing PCMs in the form of a slurry were prepared in shares of 0, 5, 10, and 15 wt %. Geopolymers were produced based on fly ash. The foaming process was carried out using hydrogen peroxide (H_2_O_2_). Physical, mechanical, and thermal properties and analysis of the microscopic microstructure were evaluated. The introduction of Micronal 28S into the geopolymer matrix is conducive to obtaining ultralight foams with a density of about 200 kg/m^3^. As the share of the PCM increases, the thermal insulating properties of the samples increase by reaching a thermal conductivity coefficient λ of 0.057 W/m*K. Simultaneously, the specific heat increases up to 1.105 kJ/kg*K. Microstructure analysis confirmed that Micronal 28S tends to agglomerate and decrease pore size. The phase change material reduces the mechanical properties of the geopolymers. However, according to the EN 998-1 standard, the conditions for the requirements to be realized by insulating materials used in construction were met by a reference sample and one containing 5 wt % of Micronal 28S. The content in this publication addresses issues in both materials science and mechanical engineering. Although other authors have conducted research using various phase change materials, this work is the first to use PCMs with the trade name Micronal 28S to produce geopolymer foams with excellent thermal insulation properties.

## 1 Introduction

Due to uninterrupted economic development manifested by the increment in the value of industry relative to other sectors of the management, there has been an increase in demand for the use of ordinary Portland cement (OPC) as a binder for concrete. Ease forming and high strength have made concrete an excellent construction material.^1,2^ However, despite the low cost of cement and the promising mechanical properties of the finished products, OPC production poses a significant issue from an ecological standpoint. It causes notable environmental pollution and also involves a considerable energy expenditure. The production of 1 ton of ordinary Portland cement generates more than 0.8 tons of carbon dioxide. CO_2_ emitted during such production accounts for about 5–7% of global anthropogenic carbon dioxide.^1,3,4^ Meanwhile, 12 billion tons of concrete are produced globally each year, of which about 1.6 billion tons of OPC are consumed.^2^ These statistics synthesize the search for alternatives that will provide environmentally friendly solutions. The denouement is a material referred to as geopolymer. Its production causes CO_2_ emissions approximately 44–64% lower than traditional cement. Moreover, the fabrication of geopolymer material involves a much lower manufacturing temperature (<100 °C) than in the case of OPC, which is in the range of 1300–1500 °C.^4^

Geopolymers are inorganic polymers that were invented by Professor Joseph Davidovits in 1972.^5,6^ These materials are obtained by chemical reaction of aluminosilicate raw materials with an alkaline activator.^7^ There are four main categories of aluminosilicate precursors, among which occur (1) clay materials, including metakaolinite, (2) fly and volcanic ash, (3) slag, and (4) industrial waste.^5,8,9^ The most commonly used alkaline activators include hydroxides (NaOH, KOH) or sodium silicate solutions.^10^ The geopolymerization process is a complex and environmentally friendly chemical reaction occurring between oxides of alkali polysilicates and aluminosilicates, resulting in the formation of Si–O–Al polymeric bonds.^6,11^ Geopolymers are characterized by excellent properties, often comparable to those of ordinary Portland cement.^12^ They are characterized by high mechanical strength, thermal resistance, and water impermeability.^13^ In addition, these materials are resistant to chemicals such as acids,^14^ sulfates,^15^ and chlorides.^16^ Geopolymers are used in many industries. According to the reports of the *World Green Building Council* and the *Global Alliance for Buildings and Construction*, the construction sector is one of the most polluting ones, generating as much as 39% of global CO_2_ emissions.^17^ Therefore, the use of geopolymers as materials for construction is of particular interest. Moreover, the functional applications of geopolymer materials include their use as fire-resistant and high-temperature materials,^18^ absorbents,^19^ catalysts,^20^ and insulating materials.^21^

The issue of thermal insulation is extremely important from the point of view of building materials. These needs are perfectly met by geopolymer material in a foamed form, which is characterized by low water absorption and low thermal conductivity.^22^ Geopolymer foams are obtained by introducing pores into the geopolymer mortar. Examples of widely used foaming agents include hydrogen peroxide,^23^ silicon,^24^ and aluminum powder.^25^ One of the most widely used blowing agents due to its high availability, low cost, and reproducible synthesis is hydrogen peroxide (H_2_O_2_).^26^ It decomposes in an alkaline solution, releasing gaseous oxygen and water, which results in the formation of pores. The concentration of the alkaline solution, as well as the degree of dissolution of Al and Si, affect the foaming reaction of H_2_O_2_^27^ Concerning Portland foam concrete, foamed geopolymers contribute to obtaining better thermal insulation properties, higher compressive strength, and lower density.^28^

The construction industry is responsible for 40% of global greenhouse gas emissions and 36% of global energy consumption. Current environmental requirements and the desire to reduce energy costs encourage activities aimed at designing energy-efficient buildings.^29^ Increasing the thermal energy storage capacity of building materials can be achieved by incorporating phase-change materials (PCMs).^30^ Materials produced in this way have excellent thermal insulation properties that meet the needs of reducing energy consumption in the construction industry.^31−33^ PCMs can store energy in the form of latent heat during phase transformation at ambient temperature.^29^ Systems involving the use of phase-change materials have reported cost savings of 42% and energy savings of 32%.^33,34^ Phase change materials come in various forms. In general, PCMs are classified according to their chemical composition into three categories: (1) organic, which can cause a change in mechanical properties, (2) inorganic, which can be chemically unstable and result in the appearance of corrosion of materials, and (3) eutectic mixtures, which can be a mixture of inorganic and organic PCMs.^33,35^ Four categories can be distinguished among the methods of incorporating PCM into building materials. The first one is microencapsulation, which involves coating droplets or particles with a continuous foil. This is intended to produce micrometer to millimeter-sized capsules referred having a core and an inorganic coating.^36^ Microencapsulation is divided into physical, chemical, and physicochemical methods. The introduction of microcapsules into cementitious materials can be expected to improve their thermal conductivity, and increase the specific heat capacity and mechanical properties.^32^ There is a wide range of shell materials available for microencapsulation. The core material is critical to the properties and applications of the microcapsule. Therefore, the choice of core material and microencapsulation method depends on the engineering requirements.^37^ For example, Ismail et al.^38^ investigated the use of Cenospheres to encapsulate biological PCM derived from refined edible vegetable oil. Chemical etching was applied to the Cenospheres and a silica-based coating was applied to seal and prevent PCM leakage from the microcapsule. The authors demonstrated the potential of Cenospheres as an inorganic coating for PCM and the possibility of incorporating silica-coated PCM microcapsules into building materials for thermal energy storage. Cunha et al.^39^ in their work investigated the temperature performance of cement mortars with the incorporation of form-stable Phase Change Materials based on PEG. The form-stable method has gained popularity in recent years due to the simplicity and cost-effectiveness of creating composites by introducing PCM into the matrix. The addition of PCM reduces energy consumption and promotes the improvement of thermal comfort. The second method of incorporating phase change materials into concrete is shape stabilization of PCMs (SSPCM), which is accomplished using a supporting material (SM). The methods for producing SSPCM include vacuum impregnation, direct absorption, and sol–gel methods. The addition of SM may contribute to changing the thermophysical properties of PCM.^32^ Another method of incorporating PCM is the absorption of liquid PCM into porous lightweight aggregates (LWA). When PCM is phase stabilized based on porous lightweight aggregates, it can have higher strength compared to other porous structures.^40^ Macroencapsulation can be mentioned as the last of the methods for effective PCM incorporation. It increases the rate of heat transfer due to increased surface area, which leads to increased thermal conductivity.^35^ Macroencapsulated materials are easier to produce which reduces their cost.^41^

In the world literature, one can find works on the use of phase change materials as additives to geopolymer materials. For example, Rahemipoor et al.^42^ conducted a study on the incorporation of PCMs into three-dimensional (3D) printed geopolymer paste in amounts of 0, 5, 10, 15, and 20%. They reported that increasing the amount of phase change materials in the geopolymer blend increases the blend’s viscosity and ductility and extends the setting time by up to 50%, which shifts the printability window. Although the addition of PCM results in a deterioration of mechanical properties, it improves the thermal performance of the geopolymer. The geopolymer paste with 20 vol % PCM had a latent heat of 10.01 ± 0.22 J/g. The thermal conductivity of the geopolymers decreased by about 4%. In contrast, in a study by Fang et al.^43^ used an artificial geopolymer material as a novel PCM carrier. The produced composites met the criterion of an insulating material. The produced material had a high absorption of 18.7–21.0% with a simultaneous high compressive strength ranging from 36.4–60.2 MPa. With the addition of PCM, the absorption value dropped to 5.50%. The flexural strength of the composite with the phase change material was 53.2–71.3 MPa. The thermal conductivity ranged from 0.510 to 0.589 W/mK, while the enthalpy of melting was about 4.29–24.74 J/g. Pilehvar et al.^44^ determined the effect of adding two types of PCM (in liquid and solid form) to geopolymer mixtures and concrete. The samples were tested below (20 °C) and above (40 °C) the melting point of PCM. The microcapsule envelope was shown to be resistant to alkaline solution. Geopolymer composites showed better performance than concrete.

Phase change materials are an excellent component for the production of geopolymer composites for the construction industry, as they serve to increase the thermal capacity of the building’s external envelope. PCMs are heat storage systems that adsorb thermal energy from the inside and release it outside the building, which can save up to 30% of energy costs.^45^ A literature review has shown that there is increasing interest in the research area of combining PCM with geopolymer materials. There are several works in which researchers have focused on the production of energy-saving materials with the addition of PCM (consisting of n-hydrocarbon paraffin that was encapsulated in a poly(methyl methacrylate) shell) and the use of fly ash as a precursor. For instance, Wang et al.^46^ used fly ash-based geopolymer mortar as a building material with minimal carbon footprint to incorporate PCM to obtain a solution for reducing energy consumption in buildings. The addition of PCM resulted in the reduction of the thermal conductivity of geopolymers. The presence of latent heat in the produced geopolymers suggests the potential use of geopolymers as an energy-saving building material. Other authors have also noted that the incorporation of PCM into building materials is an effective way to improve energy management. In the work of Wang et al.^47^ used fly ash ceramsite and paraffin as PCM with a melting point of ∼28 °C and a latent heat of ∼193 J/g to produce a composite. The researchers proved the excellent stability of PCM carried in ceramsites, which is beneficial for thermal regulation in buildings. Also, the authors’ previous work^48^ focuses on analyzing the properties of foamed fly ash-based geopolymer materials with the addition of PCM such as MicroCaps, GR42, and PX25. The authors showed the addition of PCM to the geopolymer increases the specific heat. Such materials can be successfully used as insulation materials, as they accumulate heat very well. Although other authors have conducted research using various phase change materials, this work is the first to use PCM with the trade name Micronal 28S to produce geopolymer foams with excellent thermal insulation properties. Micronal 28S can be used as a functional component in foams, building materials, and thermal management systems, for temperature regulation to improve comfort and increase energy efficiency. The purpose of this study was to produce foamed geopolymer composites prepared on the basis of fly ash modified with the addition of phase change material. PCM was introduced into the geopolymer mixture as a slurry at 0, 5, 10, and 15% by weight. Mechanical and thermal properties were tested, and the microstructure was evaluated on the produced samples. The fabricated composites have the potential to be used in the construction industry while meeting the criteria of sustainable development. The benefits of using PCM in the construction industry are significant, especially since current efforts are to reduce the energy consumption of buildings and reduce CO_2_ emissions.^49^

The innovation of the presented research results is due to several issues. First, the innovation is the PCM material used, in the form of a suspension of microencapsulated material that is specifically Micronal 28S. So far it has not been used in insulating materials such as foamed geopolymers. Another issue is the use of two thermal insulation mechanisms, which are (1) the porous structure (with a mixture of open and closed pores); (2) the addition of a phase change material that causes heat to pass through the building envelope (penetrating) to be additionally retained (captured) by the PCM material. After the partition cools down, this heat is released, but due to the insulating nature of the partition, it does not enter the room in its entire amount but is blocked (insulated) by the porous structure of the matrix material.

## 2 Materials and Methods

### 2.1 Materials

Geopolymer samples were produced using raw materials such as fly ash, sand, cement, fly ash microspheres, and surfactant. The fly ash from the Skawina Combined Heat and Power Plant (Skawina, Poland) designated as class F ash was used as a precursor. Based on the authors’ previous research, the chemical composition and particle size characteristics of the precursor are known. The range of particle diameters of the fly ash used in the study is between 1.3 and 32.5 μm, while the *D*_50_ is 22.3 μm.^50^ The precursor parameters significantly impact the geopolymerization process and the properties of the produced samples. Some of the most important parameters include the amount of amorphous phase and the particle size of the material used. They are an indicator of the reactivity of the precursor.^51^ Usually, fly ash resulting from coal combustion contains 46–50% reactive silica, and the amount of the amorphous phase is 30–80%. The higher the amount of the amorphous phase in the fly ash, the higher the degree of reactivity in geopolymers, which translates into higher mechanical strength of the parts. In addition, fly ash with a particle size of up to 45 μm is characterized by greater reactivity at early stages.^52,53^ The fly ash selected for the study has a particle size conducive to greater reactivity. In addition, it contains a high content of elements necessary for the geopolymerization process, such as aluminum oxide and silica. At the same time, it is characterized by a low calcium content. Another raw material used in the study was quartz sand (Świętochłowice, Poland). The chemical composition of fly ash and sand is presented in Table 1.

**Table 1:** Chemical Composition of Fly Ash and Sand, wt % (Based on Refs (48,54))

	chemical composition [wt %]							
raw material	SiO_2_	Al_2_O_3_	Fe_2_O_3_	CaO	MgO	TiO_2_	K_2_O	Na_2_O
fly ash	55.90	23.49	5.92	2.72	2.61	1.09	3.55	0.59
sand	90.0–90.3	0.4–0.7	max. 0.2	0.17	0.01	0.08–0.10	–	–

Calcium aluminate cement with the trade name Górkal 70 (Górka Cement, Trzebinia, Poland) acting as a stabilizer of the porous structure of the geopolymer was also used as components of the dry mix. Fly ash microspheres (TERMO-REX S.A., Jaworzno, Poland) were used, which are responsible for the formation of closed pores. Syringaldehyde powder (Merck, Taufkirchen, Germany) was used as a surfactant. An alkaline solution was chosen for the synthesis, as it has been proven^55^ that the strength of geopolymers activated with an alkaline solution is higher than that obtained from acid activation. Moreover, as shown by the authors,^56^ the use of a NaOH activator improves the compressive strength of geopolymers with clay and fly ash compared to the KOH activator. The raw materials were activated with an alkaline solution consisting of 10-molar sodium hydroxide and an aqueous solution of sodium silicate (R-145), which were mixed in a ratio of 1:2.5. 35% hydrogen peroxide—H_2_O_2_ (Grupa Azoty, Puławy, Poland) was used as a foaming agent. An acrylic microencapsulated phase change material under the trade name Micronal 28S (Microtek Laboratories, Inc., Dayton, USA) was added to the geopolymer mixture. It is used as a functional component in foams (insulation materials), foamed building materials, and materials for temperature regulation to improve thermal comfort and indoor climate. Micronal 28S occurs as a suspension (43% ± 1 solids) in white to slightly off-white color consisting of particles with a mean size of 1–5 μm. The melting point of Micronal 28S is 28 ± 2 °C and the heat of fusion is ∼140 J/g.^57^

### 2.2 Mix Design Procedure

**Figure 1:**
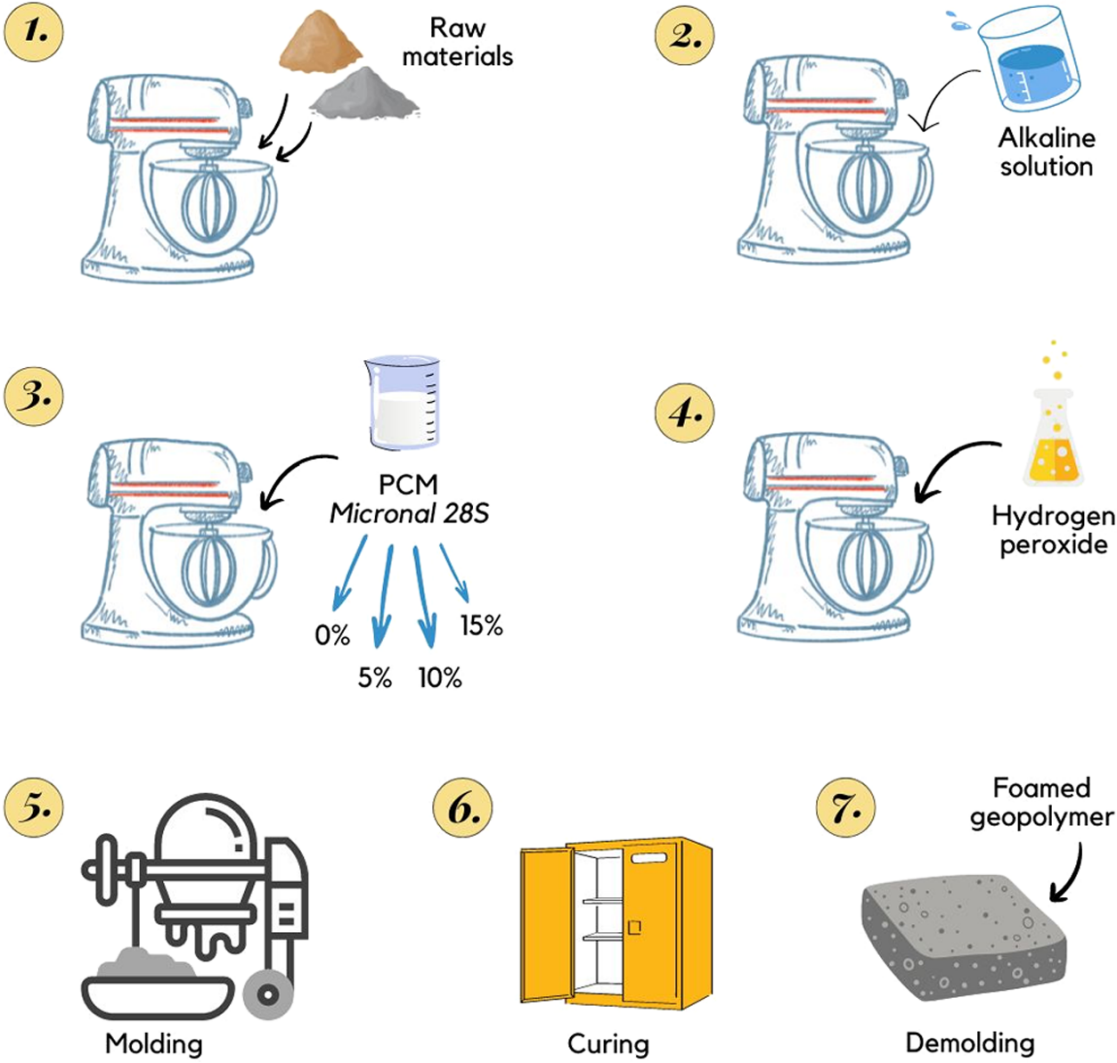
Scheme of the production process of foamed geopolymer samples with the addition of PCM.

The process of producing foamed geopolymer samples for the research conducted in this study consisted of several stages, the course of which is shown in Figure 1. The process of preparing the geopolymer mixture was carried out at room temperature. First, all dry bulk ingredients separately for each mixture (795 g of fly ash, 80 g of sand, 100 g of cement, 160 g of fly ash microspheres, and 5 g of Syringaldehyde powder) were placed in a bowl and mixed in an M/LMB-s cement mortar mixer (GEOLAB, Warsaw, Poland) for approximately 5 min to evenly combine all materials. An alkaline solution of 310 to 375 mL was then added. The amount of solution depended on the variant produced and the related amount of added PCM. For samples with a higher proportion of PCM, less solution was added to obtain mixtures of comparable consistency. The ingredients were mixed for about 10 min until a homogeneous geopolymer mixture was obtained. In the next stage, depending on the variant produced, the phase change material was introduced in the following percentages of 0, 5, 10, and 15% by weight (0, 60, 127, and 201 g, respectively) and stirred continuously for 10 min to homogenize the mixture and thoroughly distribute the PCM additive throughout the geopolymer. In the last stage, hydrogen peroxide (the same amount for each variant equal to 50 mL) was added to the obtained geopolymer mixture to acquire a porous structure of the material. After the reaction started, the foamed mixture was poured into molds. The process of venting the mass on a vibrating table was not used due to the need to obtain as many pores as possible. The prepared molds filled with geopolymer mixture were heated at 60 °C for 24 h in an SLW 750 laboratory dryer (POL-EKO Perfect-Environment, Wodzisław Śląski, Poland). Plates of 200 × 200 × 25 mm were used to measure thermal properties. Compressive strength tests were carried out on 25 × 25 × 25 mm cube-shaped specimens. The samples were tested after 28 days of seasoning at room temperature. The chemical composition of the produced geopolymer foams with the addition of phase change material is presented in Table 2.

**Table 2:** Compositions of Produced Geopolymer Foams

sample ID	fly ash [g]	sand [g]	cement [g]	microspheres [g]	syringaldehyde [g]	PCM Micronal 28S [g]	H_2_O_2_ [mL]	alkaline solution [mL]
0% M28S	795	80	100	160	5		50	365
5% M28S	795	80	100	160	5	60	50	375
10% M28S	795	80	100	160	5	127	50	350
15% M28S	795	80	100	160	5	201	50	310

## 3 Experimental Investigations and Results

### 3.1 Density

The density of the produced geopolymer foams was determined geometrically as the ratio of the sample mass to its volume. The samples were weighed on a RADWAG PS 200/2000.R2 precision balance (RADWAG Wagi Elektroniczne, Radom, Poland) with an accuracy of 0.01 g. Dimensions in three axes were measured with a digital caliper with an accuracy of 0.01 mm. The density was determined separately for each of the two plates produced. Table 3 shows the average results of density measurements of foamed geopolymer samples.

**Table 3:** Density of Geopolymer Foams with the Addition of Micronal 28S

sample ID	density [kg/m^3^]
0% M28S	228.39 ± 8.51
5% M28S	215.86 ± 4.20
10% M28S	199.25 ± 3.87
15% M28S	226.61 ± 0.30

Analyzing the obtained results, it can be observed that the highest density (228.39 kg/m^3^) is characterized by the reference sample—without the addition of phase change material (0% M28S). Up to a content of 10 wt % Micronal 28S in the sample, the density of the material decreases. For the sample with 5 wt % PCM addition, a decrease in density of about 5.5% was recorded compared to the reference sample. Similarly, the addition of 10 wt % PCM contributed to a decrease in the density of the initial sample by approximately 13%. On the other hand, the introduction of Micronal 28S at 15 wt % into the geopolymer mixture results in a redensification of the material. The density obtained for this variant is less than 1% lower compared to the reference sample. The discrepancies in material densities are probably related to a change in the consistency of the geopolymer mass due to the introduction of PCM material. The surface tension of the geopolymer mixture was changed due to which the pore formation process and stability of the produced foams were different.

### 3.2 Compressive Strength

Compressive strength tests were carried out on cube-shaped specimens. The shape and dimensions of the specimens are regulated in accordance with PN-EN 12390-7:2013-03. The strength tests were conducted under PN-EN 12390-3:2019-07 (Testing of concrete—Part 3: Compressive strength of test specimens) on an MTS Criterion 43 testing machine at a measuring range of up to 30 kN. The test rate was 10 mm/min. TestSuites 1.0 software (MTS System Corp., Eden Prairie, MN, USA) was used. Compressive strength tests were performed on samples with dimensions of 25 × 25 × 25 mm. Ten repetitions of the measurement were performed and the average value was determined. The compressive strength of the material is determined based on eq 1:
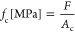
1where: *f*_c_—compressive strength [MPa], *F*—maximum load [N], and *A*_c_—area of the cross-sectional area of the specimen on which the compressive force acts [mm^2^].

**Figure 2:**
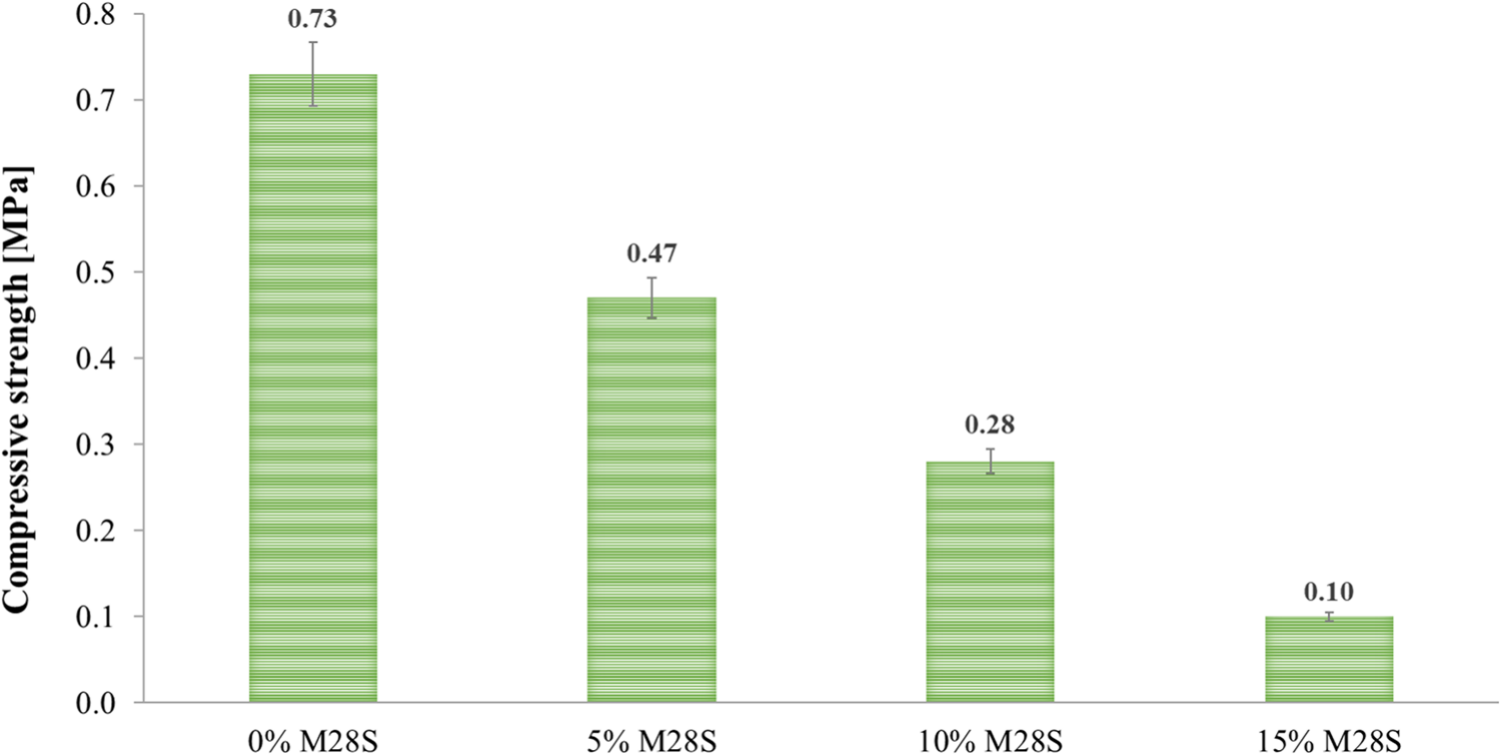
Compressive strength of foamed geopolymers with the addition of Micronal 28S PCM.

Results showing the effect of the addition of Micronal 28S phase change material on the compressive strength of geopolymer foams are presented in Figure 2.

Analysis of the results presented in Figure 2 shows a linear dependence of the strength of the geopolymer foams on the amount of PCM additive incorporated into the geopolymer paste. The compressive strength has a decreasing character as the share of Micronal 28S in the matrix increases. The reference material (0% M28S) shows quite low strength of 0.73 MPa. The introduction of 5 wt % Micronal 28S into the geopolymer paste resulted in a 36% decrease in the compressive strength of the samples compared to the material without this addition. The share of PCM in the amount of 10 wt % contributed to reducing this value by 62%. The addition of 15 wt % Micronal 28S to the geopolymer matrix causes a decrease in compressive strength by as much as 86% and generates a value of 0.10 MPa. This is a well-known phenomenon and has been reported in the literature.^58−61^ However, it should be noted that in the case of the use of foamed geopolymers as an insulating (rather than structural) material, the strengths obtained in tests are sufficient for the use of such materials and correspond to the strengths of other commonly used insulating materials, such as polystyrene, wool or polyurethane foams.

### 3.3 Thermal Conductivity and Specific Heat

**Figure 3:**
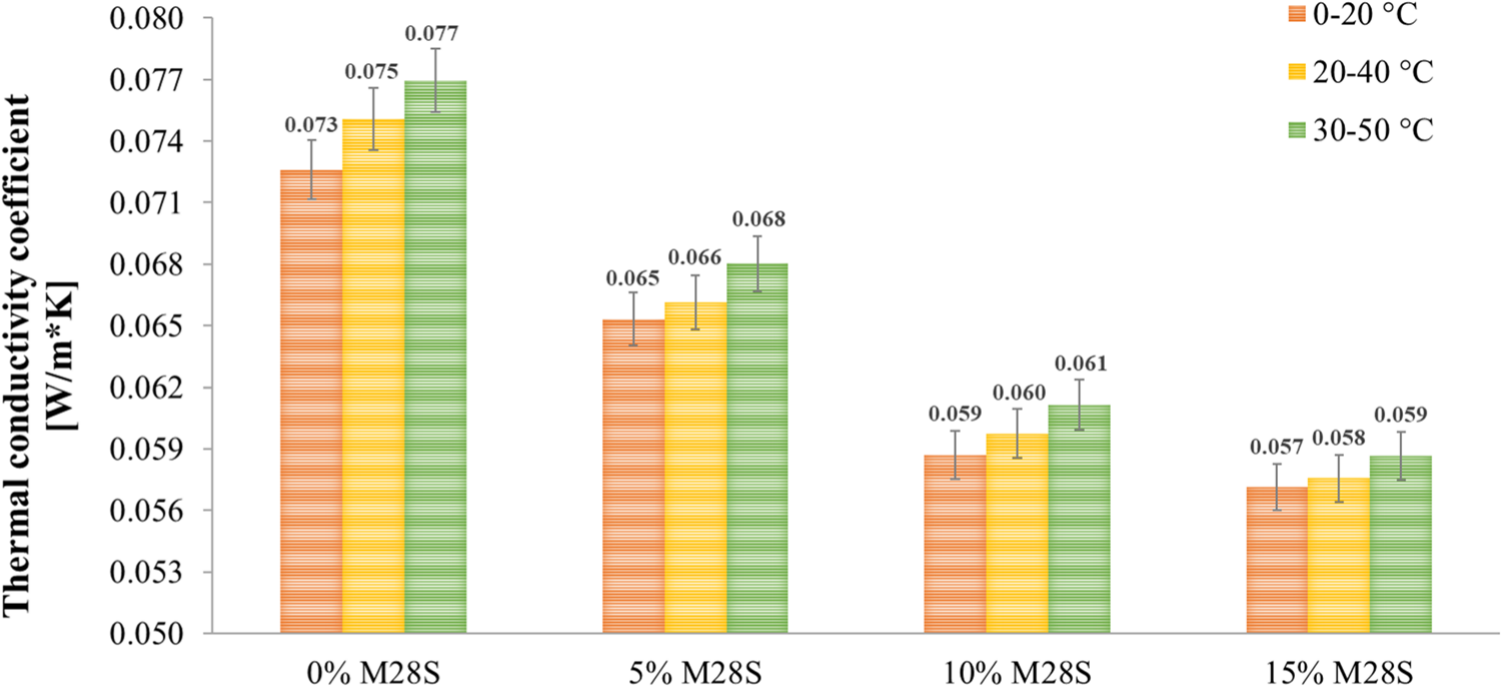
Thermal conductivity of foamed geopolymers with the addition of Micronal 28S PCM.

**Figure 4:**
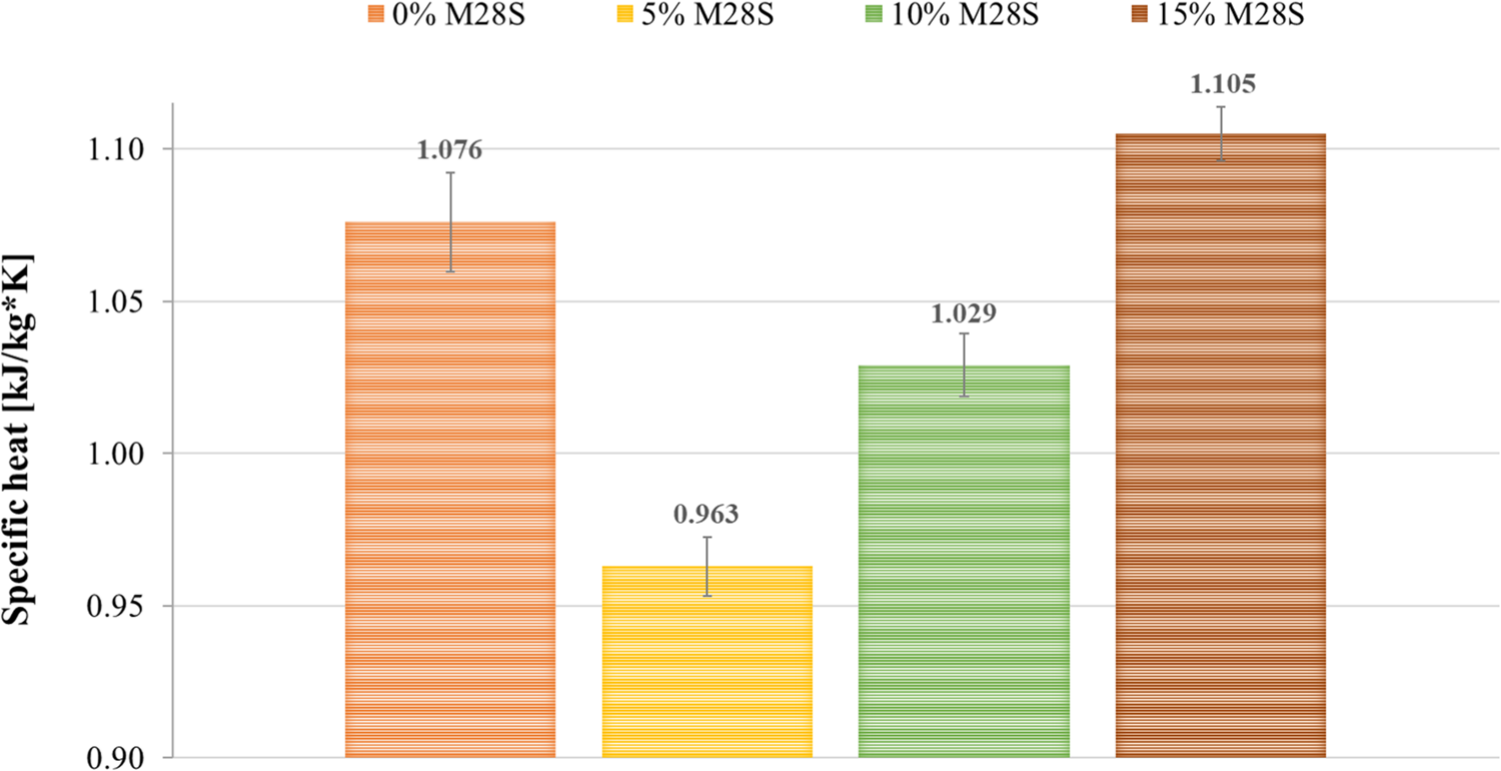
Specific heat at 27.5–32.5 °C for foamed geopolymers with the addition of Micronal 28S PCM.

Measurements of thermal conductivity coefficient (λ) and specific heat (Cp) were carried out using the HFM 446 plate apparatus (Netzsch, Hanau, Germany) operating in accordance with standards including ASTM C1784, ASTM C518, ISO 8301, EN 12664. Temperature regulation and control is verified by a Peltier system. Measurement accuracy is ±1–2%, repeatability ±0.25%, and reproducibility ±0.5%. Thermal properties were determined using the hot and cold plate method. The thermal conductivity coefficient was appointed in three temperature ranges: 0–20, 20–40, and 30–50 °C. The specific heat was determined in the temperature range of 27.5–32.5 °C. The selection of these ranges is since insulating materials can operate at temperatures above 30 °C. A graph showing the influence of the share of the introduced phase change material on the value of thermal conductivity and specific heat is presented in Figures 3 and 4, respectively. Average values were determined based on two measurements.

Based on the obtained thermal conductivity results, it was noted that the thermal conductivity coefficient increases with an increase in the test temperature by approximately 2–3% each time. The higher the test temperature, the faster the heat transfer mechanism. Generally, values in the range of 0.057 to 0.077 W/m*K were obtained. The 0% M28S reference sample has the highest thermal conductivity (0.073–0.077 W/m*K). The introduction of a phase change material into the geopolymer matrix resulted in a decrease in the thermal conductivity. The addition of Micronal 28S in the amount of 5 wt % contributed to reducing the coefficient by 11–12%. The incorporation of PCM at 10 wt % reduced the reference sample coefficient by 19–21%. The modification of the geopolymer matrix with Micronal 28S in an amount of 15 wt % resulted in a decrease in the λ coefficient by 22–23%. The lower the thermal conductivity coefficient, the better the thermal insulation properties of the material.

Analysis of the thermal test results revealed the effect of the addition of phase change material on the specific heat of the samples. The specific heat for the reference material is 1.076 kJ/kg*K. Successively increasing the share of Micronal 28S to 10 and 15 wt % causes an increase in specific heat to values of 1.029 and 1.105 kJ/kg*K, respectively. By raising the amount of Micronal 28S additive from 5 to 15 wt %, the value increases by almost 15%. The specific heat of the sample with the addition of 15 wt % PCM is about 3% higher than that of the reference material. A higher specific heat value provides a greater ability for the material to accumulate heat. Based on the results of specific heat, the volumetric heat capacity of the tested materials was calculated using also the results of the density of the samples (Table 3). The calculations resulted in the values shown below in Table 4.

**Table 4:** Volumetric Heat Capacity of Foamed Geopolymers with the Micronal 28S Additive

sample ID	volumetric heat capacity [J/kg*m^3^]
0% M28S	245.75
5% M28S	207.87
10% M28S	205.03
15% M28S	250.40

### 3.4 Determination of Pore Size Changes Using Image Analysis

**Figure 5:**
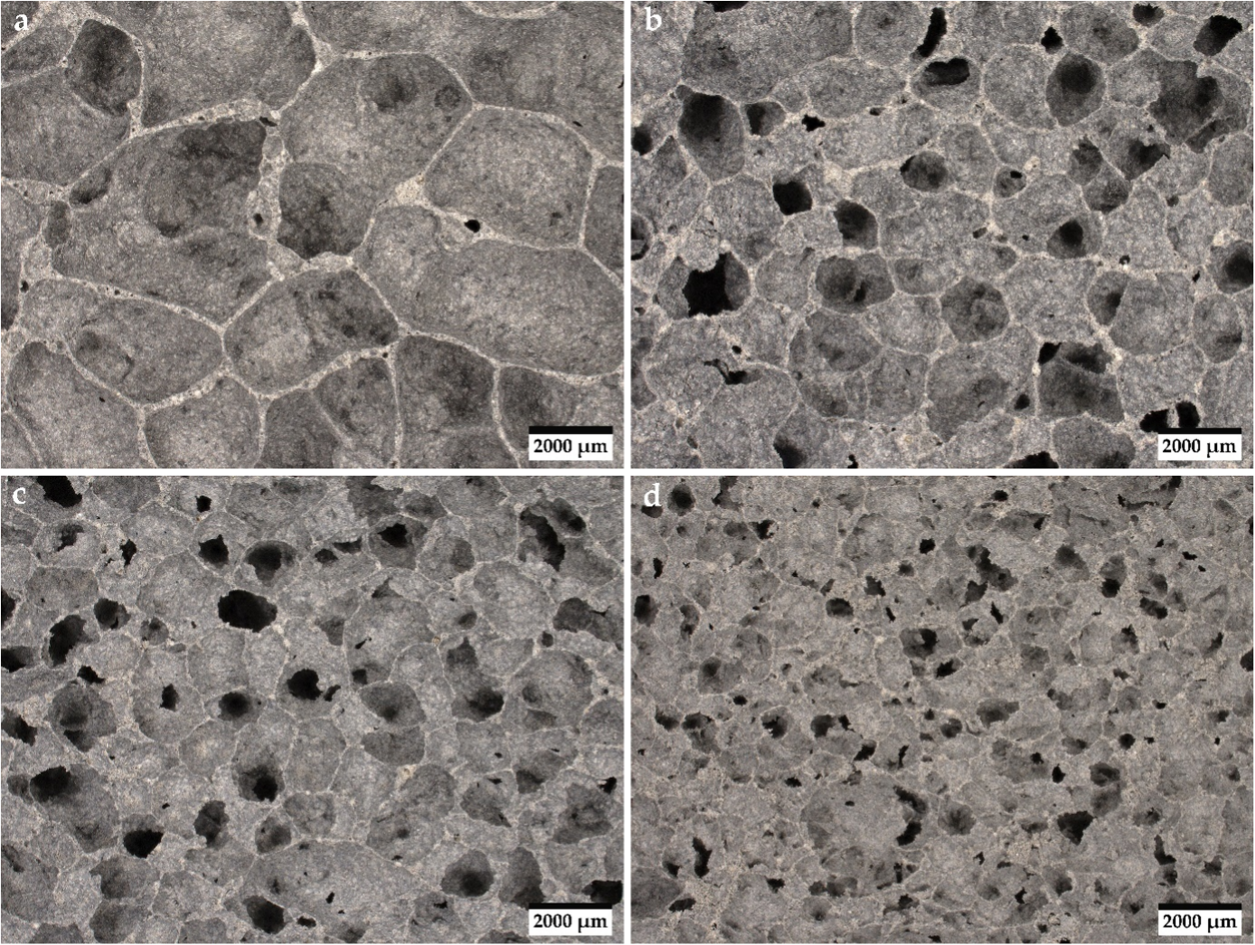
Morphology of geopolymer foams, where **a** 0% M28S, **b** 5% M28S, **c** 10% M28S, and **d** 15% M28S.

Analysis of pore size changes in the structure of geopolymer samples resulting from the amount of phase change material introduced was performed using a Keyence VHX-7000 series digital optical microscope (KEYENCE International, Mechelen, Belgium). Images of individual samples were recorded at 20× magnification and are shown in Figure 5.

Based on the image analysis, the average pore size present in the structure of the foamed geopolymer was determined. The produced foams are characterized by macroporosity, distinguished by the presence of mainly closed pores. The 0% M28S reference sample has the largest pore size among all four variants, ranging from 2000 μm to about 4000 μm. The introduction of PCM additive in the amount of 5 wt % reduced the pore size to values within the range of 1000–2000 μm. The addition of Micronal 28S in a proportion of 10 wt % causes a decrease in pore diameter to about 1000–1500 μm. The introduction of PCM in an amount of 15 wt % reduces the pore size to a diameter in the range of 500–1000 μm. The higher the percentage share of PCM addition, the finer the pores. The porous structure of geopolymer foams with increasing the amount of Micronal 28S to a value of 10 wt % shows increasing homogeneity. However, after the introduction of PCM in the amount of 15 wt %, the pores in the geopolymer structure are less regular and heterogeneously distributed.

### 3.5 Assessment of the Microstructure of Foamed Geopolymers with the Addition of Phase Change Materials

**Figure 6:**
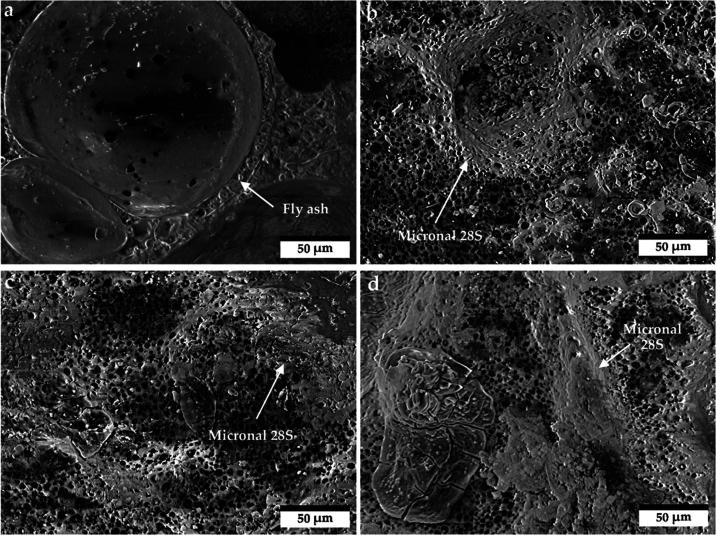
Micrographs of geopolymer foams, where **a** 0% M28S, **b** 5% M28S, **c** 10% M28S, and **d** 15% M28S.

Micrographs of geopolymer foams were taken using a JSM-IT200 InTouchScope Scanning Electron Microscope (JEOL Ltd., Tokyo, Japan). The images were created at 500× magnification. The morphology of the geopolymer structures is shown in Figure 6.

Micrographs confirm the amorphous structure of the produced geopolymer foams. The 0% M28S sample illustrated the presence of undissolved fly ash particles. Figure 6b–d show areas that indicate the presence of the Micronal 28S phase change additive in the geopolymer matrix. PCM appears in the form of brighter areas. The area indicating the presence of the additive increases with the increase in the share of Micronal 28S in the matrix. The micrographs of geopolymers with the addition of PCM also show the presence of evenly distributed very fine pores with a size of approximately 3–7 μm.

## 4 Discussion

As part of this work, the physical, mechanical, thermal, and optical (visual) properties of the produced geopolymer foams were analyzed. The results of the conducted research showed that the contribution of the introduced phase change material under the trade name Micronal 28S has a significant impact on the final properties of the produced foamed geopolymers. As the literature analysis showed, so far the authors have not used Micronal 28S as an additive for foamed geopolymer foams. This is a new issue, therefore the discussion was conducted regarding studies in which other PCMs were added to geopolymer materials.

The results obtained in this work suggest a decrease in the density of foams modified with the addition of Micronal 28S. The reference sample achieved the highest density (approximately 228 kg/m^3^). The addition of 5 and 10 wt % PCM caused a decrease in density by about 5.5 and 13%, respectively. The 15 wt % share of PCM contributed to an increase in this value, ultimately reaching a density close to that of the reference sample, at 226 kg/m^3^. Similar results were also observed by other authors. For example, Haily et al.^33^ in their work added form-stable phase change material (PU@PCM) to geopolymer mortar at 0, 5, 10, 15, and 20%. The geopolymer without the addition of PCM had a density of 2200 kg/m^3^. However, the introduction of PU@PCM to the mortar resulted in a decrease in density for the maximum share by up to 30%. As more geopolymer paste is adsorbed onto the phase change material, the viscosity of the mix increases and promotes the likelihood of trapped air voids being formed during the mixing and pouring process.^62^ In addition, the hydrophobic nature of PCM tends to repel water, and air may adhere to the microcapsules, increasing the porosity of the mixture.^63^ In general, such a reduction in density is considered beneficial as long as it does not result in a decrease in other properties.^64^

Analysis of the compressive strength of the produced geopolymer foams showed that as the share of Micronal 28S in the geopolymer matrix increases, there is a linear decrease in the strength of the samples. Increasing the PCM share by 5 wt % each time causes a decrease in this property compared to the reference sample by 36, 62, and 86%, respectively. In general, the compressive strength of the obtained composites ranged from 0.10 to 0.73 MPa. Similar results were obtained in the previous work,^48^ where the values also did not exceed 1 MPa. After the introduction of a modifier in the form of a fine white powder (PX25), the authors noted a decrease in compressive strength. Pilehvar et al.^65^ noticed that the decrease in the strength of geopolymer concrete may be caused by a weak bond between the PCM and the concrete matrix, as well as lower stiffness and strength of phase change materials, which results in cracks and deformations during the compression test. On the other hand, Marske et al.^66^ used shape-stabilized PCM in their work and obtained the compressive strength of 0.7 MPa (at 30 °C) and 1.2 MPa (at 10 °C) for the finished products, which is a sufficient value for thermal energy storage applications, e.g., for energy-saving walls. Although the materials produced in this work meet the basic requirements of the standards, there are possible methods to increase their compressive strength. In the manufactured composites, it would be possible to partially replace the PCM with sand. Sand increases the strength of the parts. The literature^60,61,67^ shows that replacing sand with microencapsulated PCM often decreases the compressive strength of the parts. Due to the low stiffness of mPCM concerning sand and the weaker bond between the matrix and mPCM, a decrease in the compressive strength of the material is observed.^68,69^ Another way to increase the strength of the samples could be to introduce an additive in the form of slag to the geopolymer matrix. Lei et al.^70^ showed that the amount of slag in the binder is a key factor in increasing the early ability of fly ash-slag geopolymer mortar to gain strength.

Thermal properties tests confirmed that the thermal conductivity coefficient of the reference sample is 0.073–0.077 W/m*K, while the geopolymers in the addition of Micronal 28S range from 0.057 to 0.068 W/m*K. The higher the temperature used during the test, the higher the thermal conductivity coefficient of the samples, which is associated with faster heat transfer. The introduction of PCM into the geopolymer matrix resulted in a decrease in the thermal conductivity coefficient. Accordingly, with the increase in the share of Micronal 28S to 5, 10, and 15 wt %, a decrease in the coefficient was recorded by 11–12, 19–21, and 22–23%, respectively. The measured values meet the RILEM criterion (0.75 W/m*K), which means that the samples produced are insulating materials.^71^ Higher thermal conductivity of materials is required to accelerate the phase-changing process, while lower conductivity of the building envelope is conducive to reducing heat transfer.^30,72,73^ In the previous work,^48^ the lowest thermal conductivity coefficient was obtained for geopolymers with a content of 10 wt % the addition of MicroCaps, as well as with the addition of 5 wt % GR42 and it was approximately 0.070 W/m*K. Dora et al.^74^ in their work studied the effect of PCM addition on the properties of foam concrete slabs. The introduction of PCM into the geopolymer matrix increased the number of pores in the material, making it less dense. This resulted in a lower density of the material and lower mechanical strength. Due to the presence of empty voids, the thermal conductivity of the fabricated panels decreased from 0.26 to 0.08 W/m*K when 10 wt % of PCM was introduced into the matrix.

Comparing the results obtained to studies conducted on concrete containing different percentages of PCM, it can be concluded that the thermal performance of the geopolymer foams produced in this work was significantly higher. Hunger et al.^75^ observed a decrease in thermal conductivity and an increase in heat capacity by incorporating PCM into the material, which improves the thermal properties of concrete and thus promotes saving energy. The thermal conductivity of the materials produced in this work was almost 97–98% lower than that of concrete samples, which confirms their significantly better thermal performance. Also referring to the results obtained for geopolymer materials with the addition of PCM and described in the literature,^42^ it can be noted that the inclusion of a phase change material in the geopolymer matrix significantly improves the material’s thermal performance. The thermal conductivity of these materials remained around 0.5 W/m*K. In contrast, the authors of this work obtained a thermal conductivity coefficient about 8 times lower. This is also confirmed by the results obtained in the work of Bąk et al.,^48^ where thermal conductivity values of 0.07 W/m*K were obtained for fly ash-based geopolymers with the addition of PCM. This unambiguously indicates that the material produced within the framework of this work is characterized by significant thermal performance compared to other concrete materials presented in the literature.

Significantly, the heat storage is not equivalent to insulation. However, PCMs encapsulated inside geopolymer composites can provide very effective thermal insulation and energy efficiency. Such studies were conducted by, among others, Fateh et al.,^76^ who studied the effect of PCM on insulation and obtained optimal effects when a PCM layer was placed inside. PCM materials use the ability to store and release latent heat of phase change with a small temperature change. Gu et al.^77^ also conducted studies on the effect of foams with the addition of PCM microcapsules on the thermal insulation properties of cement. The authors showed that, compared to foamed cement, the cementitious foam material with PCM increased the cement’s ability to store heat while improving its insulating properties. This provides excellent opportunities for the application of this material in the construction industry.

The analysis of the results obtained by measuring specific heat showed that the reference geopolymer has this value at 1.076 kJ/kg*K. The introduction of 5 wt % PCM into the matrix reduces the specific heat of the sample by approximately 11%. However, increasing the share of PCM in amounts of 10 and 15 wt %, respectively, favors a linear increase in this value up to 1.105 kJ/kg*K. A similar relationship for the same shares of PCM addition, although higher values of specific heat were obtained, was registered in a previous work.^48^ Also, Cao et al.^78^ observed an increase in the heat capacity of samples with increasing PCM concentration. The specific heat of geopolymer concretes increases when the concentration of microcapsules increases, which may be due to the lower specific heat of geopolymer concrete with respect to PCM.^62^ Similarly, Shadnia et al.^79^ as a result of the research conducted as part of their work, noted an increase in the specific heat of the geopolymer mortar after the introduction of PCM. The phase change material can therefore effectively reduce heat transport through the geopolymer material.

The produced geopolymer foams show a decrease in pore size with an increase in the addition of PCM in the geopolymer matrix. For a 15 wt % share of Micronal 28S, a tendency to form less regular pores was observed. Meng et al.^80^ in their work noted that a 20% PCM addition would destroy the pores of the foamed cement.

Microstructure analysis of the produced geopolymer foams revealed the absence of cracks in the matrix. However, there is a noticeable tendency toward agglomeration of Micronal 28S. A similar behavior of PCM in a geopolymer sample was observed in the work of Rebelo et al.^81^

According to the European standard EN 998-1,^82^ thermal conductivity should be less than 0.2 W/m*K, and compressive strength should be in the range of 0.4 to 5 MPa. Often, higher thermal insulation of a material is associated with a decrease in its mechanical strength, which is a significant challenge when designing the composition of the produced foams^83^ Tests carried out within the framework of this work allowed to prove that the addition of Micronal 28S with a share of 5 wt % meets the requirements of the EN 998-1 standard and ensures higher thermal insulation properties compared to the reference material.

Moreover, the latent heat of phase transformation is a very important feature of composites containing PCM. Large values of this parameter distinguish such composites from traditional building materials. Phase change materials are characterized by high values of latent heat of phase transformation, which is as high as 250 kJ/kg for homogeneous materials (pure PCMs), while for microencapsulated materials it is about 100 kJ/kg.^84^ The materials used in the study (Micronal 28S) have a latent heat of phase transformation value of 140 J/g. The entire group of materials produced by CAVU Group designated as MICRONAL is characterized by a latent heat of phase transformation (Δ*H*_m_ in the range of 130–160 kJ/kg).^85^ The durability of microencapsulated PCMs and specifically Micronal with similar parameters to the one described in the article was studied in the paper.^86^ The authors examined three different PCMCs, including those coated with metal. The study showed that Micronal PCM microcapsules exhibit better heat storage/release properties than other microcapsules, with no significant changes in their thermal behavior. DSC analysis shows that Micronal microcapsules exhibit a slight reduction in heat storage capacity and retain good morphology from the SEM image. The PCM material used by the authors^87,88^ is microencapsulated in an acrylic polymer shell. These materials are characterized by nontoxicity, ease of preparation, good thermal stability and chemical resistance. The use of acrylic polymer shells has also been studied by other authors,^89^ and it was shown that the maximum percentage of total fatty acids in the stabilized PCM was 70% by weight, with no leakage of fatty acids observed when the mixtures were heated above the melting points of the fatty acids.

Based on the literature review, it can be concluded that PCM materials microencapsulated in an acrylic polymer shell are characterized by high thermal and mechanical stability protecting the active material—phase change material from flowing out. This is one of the best solutions when it comes to PCM encapsulations. The test material used by the authors in the form of Micronal 28S should also be considered as a material resistant to leakage and degradation both thermally and mechanically. Considering the application of this material, the temperature range of its use as well as exposure to mechanical damage, it should be considered that there is no potential risk for these materials to degrade.

The proposed geopolymer foams with PCM can be successfully used as energy-efficient thermal insulation materials, among others due to the use of coal combustion byproducts (fly ash) for its production. The process of manufacturing geopolymers is also energy-efficient, consuming 4 to 8 times less electricity than in the case of production using traditional concrete. Phase change materials also improve the heat storage capacity of buildings, reducing energy losses. Foamed geopolymer composites are more environmentally friendly, so they can successfully replace other insulating materials in construction. Due to their ability to store heat and good thermal conductivity coefficient parameters, such materials provide greater thermal comfort concerning buildings in which traditional solutions were used. Such materials can be used in the housing of thermal installations, which can insulate the installation from the environment and at the same time prevent it from overcooling. The produced geopolymer foams with the addition of PCM can be used in construction as external or internal thermal insulation of buildings. Compared to traditional thermal barriers used in construction (such as e.g., styrofoam), the developed materials provide greater thermal comfort through good parameters of the thermal conductivity coefficient and heat storage capabilities. The results obtained in the research conducted in this work provide a solid basis for further analyses in terms of application in industry.

## 5 Conclusions

The main aim of the present study was to analyze the effect of the addition of a phase change material under the trade name Micronal 28S on the density, compressive strength, and thermal properties of the produced geopolymer foams. Mixtures containing PCM at 0, 5, 10, and 15 wt % were prepared. Based on the research results, the following conclusions were formulated:

(1) The reference material has the highest density (228 kg/m^3^). As the PCM share increased to 5 wt % and then to 10 wt %, the density of the foams decreased successively by 5.5 and 13%. However, the introduction of an addition of 15 wt % to the geopolymer matrix promotes the density of the material. The addition of phase change materials affects the consistency of the mixture and the stability of the foamed structures obtained.
(2) A higher share of Micronal 28S in the geopolymer mixture results in a decrease in the compressive strength of the produced foams. The use of PCM in the amount of 5, 10, and 15 wt % resulted in a reduction of mechanical properties by 36, 62, and 86%, respectively, compared to the reference sample (0.73 MPa).
(3) As the share of Micronal 28S in the geopolymer matrix increased to 5, 10, and 15 wt %, there was a decrease in the thermal conductivity coefficient by 11–12, 19–21, and 22–23%, respectively. For the reference sample, the value of the thermal conductivity coefficient was approximately 0.073–0.077 W/m*K, while the foams modified with Micronal 28S obtained values ranging from 0.057 to 0.068 W/m*K. The materials produced are insulating materials. Their insulating properties are higher as the share of PCM in the matrix increases. Obtaining a thermal conductivity coefficient of 0.057 W/m*K is undoubtedly a great success, and very rarely are similar values obtained for geopolymer materials. This achievement represents somewhat of a breakthrough in the development of foamed insulating structures based on geopolymer materials.
(4) The specific heat of the produced geopolymer foams was approximately 1 kJ/kg*K. For the reference sample, this value was 1.076 kJ/kg*K. Introduction 5 wt % PCM into the mixture resulted in a decrease in the specific heat of the geopolymer by about 11%. However, increasing the share of Micronal 28S to 10 and 15 wt % resulted in an increase in specific heat to 1.105 kJ/kg*K. This is the highest value obtained among all variants produced. The introduction of phase change material into the geopolymer matrix effectively limited heat transport through the geopolymer.
(5) The proportion of Micronal 28S additive affects pore size. The higher the share of PCM in the matrix, the smaller the pores of geopolymer foams.
(6) Microstructure analysis confirms that Micronal 28S tends to agglomerate. No cracks were observed in the geopolymer matrix.

Summarizing the test results obtained for all analyzed geopolymer foams, it can be concluded that the addition of phase change material under the trade name Micronal 28S causes a decrease in density, strength properties, and thermal conductivity coefficient. According to the EN 998-1 standard, insulating materials used in construction should have a thermal conductivity coefficient λ of a maximum value of 0.2 W/m*K and a compressive strength of ≥0.4 MPa. In accordance to this standard, all obtained materials can be described as thermal insulating, but the mechanical strength requirements are not met by samples with the addition of Micronal 28S in the amount of 10 and 15 wt %. Geopolymer foam modified with the addition of Micronal 28S in a share of 5 wt % allows the improvement of the thermal insulating properties to the reference material while ensuring sufficient strength of the plate, which qualifies this variant for potential use in construction.

The present work confirms that it is possible to produce completely noncombustible insulating materials using phase change materials. Given the significant thermal resistance of geopolymers, they can be used in many applications where energy-efficient solutions are required.
